# Organotypic brain slices: a model to study the neurovascular unit micro-environment in epilepsies

**DOI:** 10.1186/2045-8118-10-11

**Published:** 2013-02-07

**Authors:** Mélanie Morin-Brureau, Frédéric De Bock, Mireille Lerner-Natoli

**Affiliations:** 1 Institut de Génomique Fonctionnelle, CNRS UMR5203, INSERM U661, Université Montpellier 1, 2, Montpellier, France; 2 Department of Neurosurgery, Thomas Jefferson University, 1020 Locust Street, JAH 454, Philadelphia, PA, 19107, USA

**Keywords:** Neurovascular unit (NVU), Blood–brain barrier (BBB), Organotypic brain slices, Epilepsy

## Abstract

**Background:**

It is now recognized that the neuro-vascular unit (NVU) plays a key role in several neurological diseases including epilepsy, stroke, Alzheimer’s disease, multiple sclerosis and the development of gliomas. Most of these disorders are associated with NVU dysfunction, due to overexpression of inflammatory factors such as vascular endothelial growth factor (VEGF). Various *in vitro* models have been developed previously to study the micro-environment of the blood–brain barrier (BBB). However none of these *in vitro* models contained a complete complement of NVU cells, nor maintained their interactions, thus minimizing the influence of the surrounding tissue on the BBB development and function. The organotypic hippocampal culture (OHC) is an integrative *in vitro* model that allows repeated manipulations over time to further understand the development of cell circuits or the mechanisms of brain diseases.

**Methods/design:**

OHCs were cultured from hippocampi of 6–7 day-old Sprague Dawley rats. After 2 weeks in culture, seizures were induced by application of kainate or bicuculline into culture medium. The regulation of BBB integrity under physiological and pathological conditions was evaluated by immunostaining of the main tight junction (TJ) proteins and of the basal membrane of microvessels. To mimic or prevent BBB disassembly, we used diverse pro- or anti-angiogenic treatments.

**Discussion:**

This study demonstrates that NVU regulation can be investigated using OHCs. We observed in this model system an increase in vascularization and a down-regulation of TJ proteins, similar to the vascular changes described in a chronic focus of epileptic patients, and in rodent models of epilepsy or inflammation. We observed that Zonula occludens-1 (ZO-1) protein disappeared after seizures associated with neuronal damage. In these conditions, the angiopoeitin-1 system was down-regulated, and the application of r-angiopoeitin-1 allowed TJ re-assembly. This article demonstrates that organotypic culture is a useful model to decipher the links between epileptic activity and vascular damage, and also to investigate NVU regulation in diverse neurological disorders.

## Background

Homeostatic maintenance is essential for proper cerebral function. The blood vessel- and non-vascular cells (neurons and glial cells) in the brain form the neurovascular unit (NVU) [1]. The NVU plays an important role in brain maintenance via cellular interactions between microvessels and parenchyma. Under physiological conditions, the NVU regulates nutrient supply, vascular growth, hemodynamics, toxin elimination and brain protection. Adherens junctions (AJs) and tight junctions (TJs) reduce the paracellular flux across brain endothelium, whereas specific transporters and receptors carry glucose, amino-acids, nucleosides, organic anions, large amino-acids, transferrin, lipo-proteins and drugs into the brain. Conversely, pathological stimuli that increase blood–brain barrier (BBB) permeability perturb brain homeostasis. The leakage of ions, water, and serum proteins into the parenchyma modifies oncotic pressure and ionic concentrations, while leukocyte extravasation triggers immune and inflammatory responses. This imbalance leads to abnormal neuronal activity or toxicity. In excitable brain structures such as the hippocampus and cerebral cortex these features induce seizures. In several CNS structures, increased BBB permeability participates in or worsens neurological disorders like Alzheimer’s disease, multiple sclerosis or chronic epilepsy [2-5].

Modeling the NVU *in vitro* has furthered the understanding of selective mechanisms which regulate permeability, toxin elimination, nutrient supply, brain protection and homeostasis regulation. Several *in vitro* cell-based BBB models have previously been developed but were unable to fully recapitulate all known features of the BBB [6,7]. Despite the conservation of endothelial cell properties *ex vivo,* their isolation from multicellular blood vessels is methodologically difficult [8]. The endothelial cell monolayer is one of the most commonly used *in vitro* models; however, it only represents a simplified view of the BBB. This simplification reduces the interactions with other cell types, which are essential for BBB maintenance [9,10]. The co-culture of astrocytes and endothelial cells is the most validated cell-based BBB model. This model contains TJs, transporters, ionic channels and high transendothelial electrical resistance (TEER) necessary for a suitable model. However the absence of other cell types such as pericytes is a limitation in dynamic studies of the NVU, including vasomodulation [11]. To counteract the lack of pericytes, the tri-culture has been developed using endothelial cells, pericyte and astrocytes cell lines. In this system, all cell types are necessary for the adequate localization of TJs and transporter functions [12]. This model can be modified depending on the research objectives, using leukocytes or neurons as the third cell type [13,14]. The tri-culture is currently one of the most representative *in vitro* models to study BBB regulation in humans [15].

Clearly, BBB models should contain most or all cellular and molecular players of the NVU and take into account the different environmental factors. Thirty years ago, Gähwiler *et al.* described an integrative model to study interactions between cell types in brain slices maintained in culture [16]. This model was simplified by growing organotypic brain slices on a membrane surface [17]. These slices maintain all cell types and their interactions for 2 weeks and were mainly used to study the activity of neural cells under diverse physiologic and pathologic conditions [18,19].

In 2003, it was shown for the first time that, despite the absence of blood flow in organotypic cortical slices, microvessels were present and able to respond to angiogenic stimuli like acidosis or hyperthermia [20]. Furthermore, microvessels preserved within organotypic slices respond to experimental seizures. We have used this *in vitro* model to study the effects of seizure-like activity on the NVU. We chose slices of rat hippocampus, since the corresponding structure in the human brain is involved in temporal lobe epilepsy (TLE). We found that kainate-induced epileptiform activities induced vascular changes in organotypic slices including angiogenesis and BBB alteration, similar to those reported in human intractable TLE and *in vivo* models [21,22].

## Methods/ design

### Organotypic brain slices

Organotypic hippocampal slices (OHCs) were prepared and cultured according to Stoppini *et al.*[17]. The brains of 6–7 day-old Sprague Dawley rats were removed after cold anesthesia and hippocampi were rapidly dissected under aseptic conditions in a dissection medium containing 50% HBSS, 50% Opti-MEM, penicillin 25 units/ml, streptomycin 25 μg/ml (Life technologies, Grand Island, NY, USA). Then transverse sections (400 μm) were obtained using a tissue chopper. Ten slices were placed on a 30 mm porous membrane (Millipore, Billerica MA, USA) and kept in 100 mm diameter Petri dishes filled with 5 ml of culture medium composed of 25% heat-inactivated horse serum, 25% HBSS, 50% Opti-MEM, penicillin 25 units/ml, streptomycin 25 μg/ml (Life technologies). Cultures were maintained in a humidified incubator at 35°C and 5% CO_2_. One week later, cultures were transferred in defined medium composed of 25% B27 supplemented neurobasal medium, 25% HBSS, 50% Opti-MEM, penicillin 25 units/ml, streptomycin 25 μg/ml (Life technologies). All animal procedures were conducted in accordance with the European Communities Council Directive of November 24, 1986 (86/6 09/EEC), and approved by the French Ministry of Agriculture (Authorization No. 34178, ML-N).

### Induction of “in vitro seizure”

After 2 weeks, membranes were transferred to 6-well plates, each well filled with 1 ml defined culture medium. To induce seizures, slices were treated with 25 μM kainate (Sigma-Aldrich, Saint-Louis, MO, USA) for 1 h or with 10 μM of bicuculline (Sigma-Aldrich) for 10 min. Control slices received no treatment. Slices were then transferred in a bicuculline-free or kainate free-defined culture medium during the recovery period (2, 12 and 24 h).

### Treatments

#### Recombinant proteins

The rat recombinant vascular endothelial growth factor (rrVEGF, R&D systems, Minneapolis, MN, USA) was added into culture medium for 24 h at 2 ng/ml. The rhAngiopoeitin-1 (rhAng-1, R&D systems) was added 4 h after seizure induction for 24 h at 400 ng/ml. LPS (100 ng/ml, Sigma-Aldrich) was added to the culture medium for 24 h.

#### Morphological study of vascularization and tight junctions

##### Immunostaining

Slices were fixed in 4% PFA for 30 min and stored at 4°C in PBS containing 0.1% NaNO_3_. To evaluate vascular density and zonula occludens-1 (ZO-1) expression, immunohistochemistry was carried out on free-floating whole slices. After pre-incubation in a PBS solution containing 10% goat serum and 0.1% Triton for 2 h at room temperature, slices were incubated for 48 h at 4°C with mouse or rabbit anti-laminin (Chemicon, Temecula CA, USA, 2E8, 1/3000 or Sigma-Aldrich, L9393 1/4000, rabbit anti-ZO-1 (Zymed, San Fransisco, CA, USA, 61–7300 1/200), Goat anti-VEGF (Santa-Cruz, Santa Cruz, CA, USA, Sc-1836, 1/200), rabbit anti-VEGFR-2 (Santa-Cruz, sc-504, 1/200), mouse or rabbit anti-GFAP (Dako, Glostrup, Denmark, 6F2, 1/1000) and mouse anti-neuN (Chemicon, MAB377,1/500). After 3 washes in PBS 1X, slices were incubated for 2 h at room temperature with secondary fluorophore-coupled antibodies against goat, mouse or rabbit. After 3 washes of 10 min in PBS 1X slices were mounted with Mowiol. For vascular density, sections were observed with a Leitz DMRB microscope (Leica, Wetzlar, Germany) equipped for fluorescent microscopy. Images were digitized by a 1392 × 1040 resolution cooled CCD camera (Cool Snap, Princeton Instrument, Trenton, NJ, USA) on a computer with Cool Snap software and transferred to Adobe Photoshop Elements (version 4) for image processing. For ZO-1, VEGF and VEGFR-2 immunostaining staining sections were observed using a confocal microscope (Zeiss 510 Meta, Göttingen, Germany) equipped with an x63 objective (oil, numeric opening 1.4). We used an argon laser (excitation 488, emission 505–530 nm) for alexa 488, a helium laser (excitation 543, emission 585–615 nm) for Texas red and a krypton-argon laser (excitation 647 nm, emission 660-700 nm) for alexa 647. Images were collected sequentially to avoid cross-contamination between fluorochromes. A series of 15 optical sections was projected onto a single image plane and scanned at 1024 × 1024 pixel resolution.

#### Quantification of vascular density

We used the point-counting method to quantify and compare the vascular density under different conditions [23]. This method has been already validated in human tissue, *in vivo* and in organotypic cultures [20-22]. It takes into account the number, size and tortuosity of vessels to characterize pathological angiogenesis. Briefly, a 5 × 5 grid was superposed onto the digitized image and the number of labeled vessels crossing the grid lines was counted. The score was expressed in arbitrary units of vascular density for a 1 mm^2^ area. Statistical analysis was performed: one-way analysis of variance (ANOVA) followed by Fisher test for OHCs (*p*<0.05 is significant).

#### Quantification of branching

To evaluate the branching after each treatment, we selected magnifications of 0.5 mm^2^ areas in the two main hippocampal fields: CA1 and CA3. Vessel branch points resulting from microvascular sprouting [24], were counted manually and results were expressed as a percent of controls. Statistical analysis was performed by one-way analysis of variance (ANOVA) followed by Fisher test for OHCs (*p*<0.05 is significant).

#### Protein expression and activation

Proteins were prepared according to our previous publication [21]. Protein samples (40 μg) boiled in Laemmli buffer containing 2-β-mercaptoethanol were loaded onto a NupageNovex 4-12% Bis-Tris Midi gel (Life Technologies), separated electrophoretically and transferred to polyvinyldifluoridine membranes (Hybond-C-extra, Amersham Biosciences, UK). Membranes were incubated overnight at 4°C with primary antibodies raised against VEGF (Santa Cruz, Sc-1836 1/200), VEGFR-2 (Abcam, Cambridge, MA, USA, Ab2349 1/1000), VEGFR-2P (Y1054 & Y1059) (Abcam, Ab5473 1/1000), ZO-1 (Zymed, 61–7300 1/800), claudin-5 (Life Technologies, 34–1600, 1/400), occludin (Life Technologies, 71–1500, 1/500), or actin (LabVision, Fremont, CA, USA, ACTN05 1/1000), then with HRP secondary antibodies against rabbit, goat or mouse IgG for 1 h at RT. Bands were visualised by chemoluminescence detection (Western Lightening, Perkin Elmer, MA, USA). Western blots were analyzed by densitometry using Photoshop and ImageJ and normalized with actin. Statistical analysis was performed by Kruskall-Wallis test, p<0.05 is significant.

#### Cytokine array

The profile of cytokines released into the culture medium was analyzed by proteome profiler using rat cytokine array (R&D Systems, Minneapolis, MN, USA, # ARY008) according to the manufacturer’s protocol. Results were analyzed by densitometry using ImageJ. For heatmap analysis, a difference between optical density of control slices and treatment conditions was calculated. Then the heatmap was generated using the MeV software (Boston, MA, USA).

## Results

### Organotypic cultures: a tool to decipher the mechanisms of BBB failure in epileptic disease

Since empty vessels respond to angiogenic factors, we studied the vascular remodelling after epileptic seizures using OHCs [20,25,26]. Previously, we observed the presence of angiogenesis and BBB permeability in pharmacoresistant temporal lobe epilepsies [22]. To determine if vascular remodeling was induced by seizures *per se*, or by cell damage or inflammation associated with severe seizures, we evaluated the vascular density in the following conditions: 1) seizures without damage induced by bicuculline; 2) inflammation induced by LPS; 3) seizures with neuronal death and inflammation induced by kainate.

**Figure 1:**
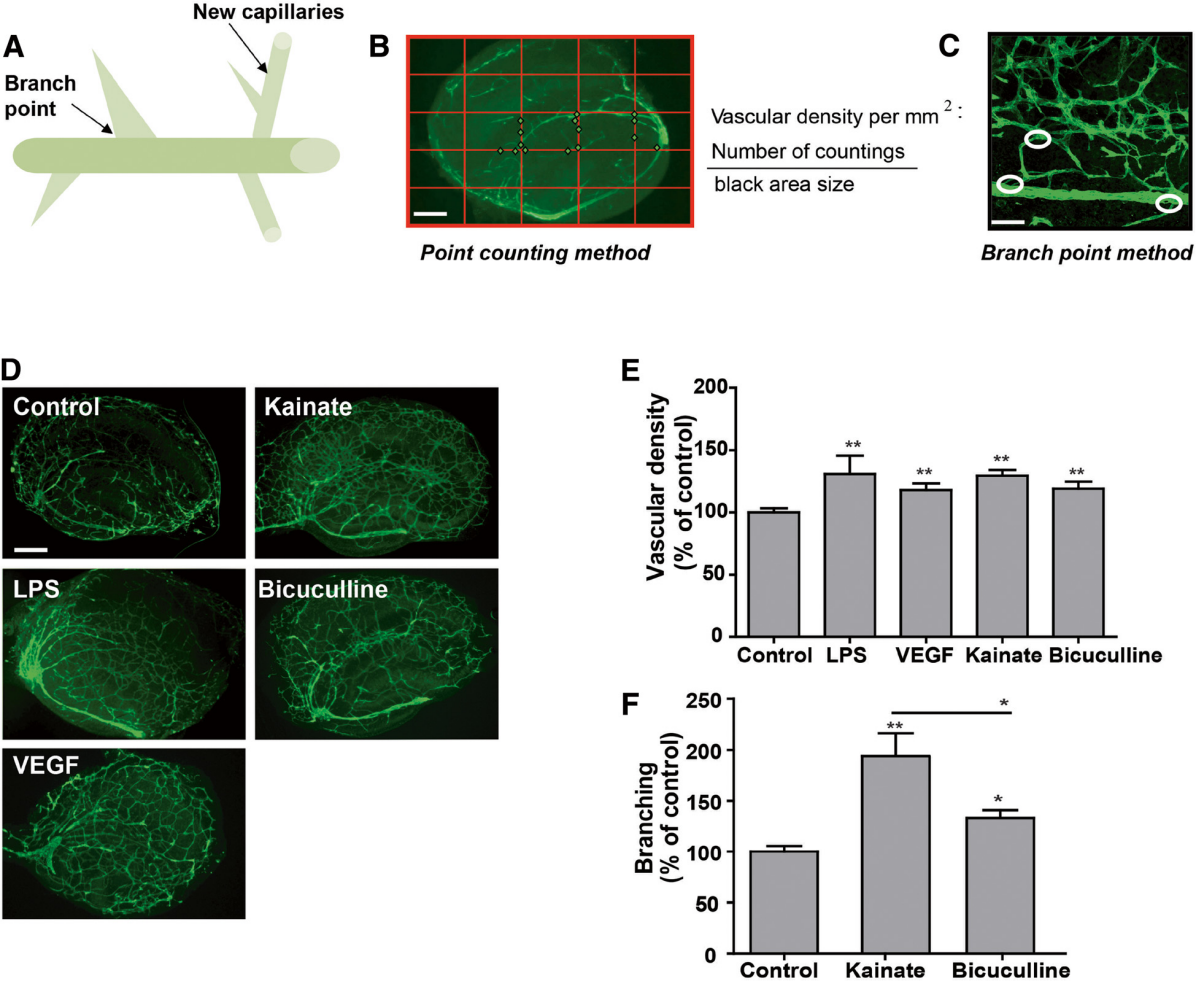
**Blood vessel responses to seizure-like activity in organotypic hippocampal culture.** (**A**) Schematic diagram of a blood vessel during the angiogenic process. Angiogenesis is the formation of a new blood capillary from the existing one. The branch point is essential for the guidance and the creation of new blood vessels. (**B**) For the point-counting method, blood vessels are stained with laminin, a specific marker of basal membrane (green). A grid is placed on the picture and the number of crossings of one vessel on the grid is counted. Therefore, the vascular density evaluation takes into account the length, tortuosity and diameter of blood vessels. For organotypic cultures, the vascular density is calculated per mm^2^. Scale Bar: 400 μm. (**C**) For the branch counting method, all branch points (circles) are manually counted. Scale bar: 50 μm. (**D**) Photomicrographs of laminin expression (green) in control slices and in slices 24 h post-treatment with kainate, LPS, bicuculline or VEGF. Scale bar: 400 μm. The vascular density increased in all conditions compared to control slices. (**E**) Quantification of vascular density. Results are expressed in % of control. ** *p*<0.01 (**F**) Quantification of branching 24 hours after seizures induced by kainate or bicuculline. The increases in branching are significant at 24 h post seizure,* p<0.05, ** *p*<0.01.

To evaluate pathological angiogenesis, we measured vascular density and branching (Figure 1A). As a positive control, we also evaluated OHCs treated with VEGF. For the “point-counting” method, the number of “crossings” was counted and normalized to slice surface area in mm^2^. The *“*branch point*”* was evaluated after magnifying the slices (Figure 1B, C). We evaluated the branching and the vascular density 24 h post-treatment. A significant increase in vascular density was observed in all conditions indicating that seizures and also inflammation are sufficient to induce a vascular remodeling, *p*<0.01 compared to control (Figure 1D, E). However laminin staining showed an increased vascular network in CA1 and CA3 areas after kainate treatment compared to other conditions (Figure 1D). Neuronal death also occurred in these two areas after kainate treatment [27]. We quantified the branching in CA1 and CA3 areas 24 h after kainate and bicuculline treatments. An increase of branching was observed in all conditions. Interestingly the branching was significantly higher in the case of seizures associated with neuronal death (kainate) than seizures alone (bicuculline), *p*<0.01 and 0.05, respectively, compared to control (Figure 1F).

We found an increase in vascular density and branching after *in vitro* seizures or inflammation, similar to previous results from rodent models or human tissue [21,22]. However, depending on the presence or absence of neuronal death, the vascular remodeling appeared to be different with an increase in branching in lesion areas. In the following study we compared changes in the NVU between the kainate and bicuculline models.

### Roles and modifications of NVU cells in pathological conditions

**Figure 2:**
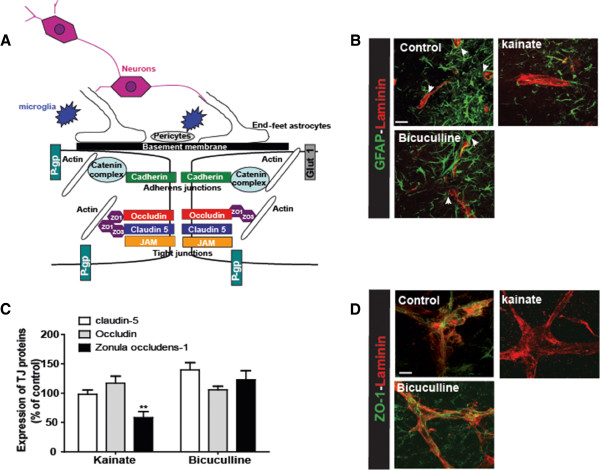
**Blood brain barrier integrity in organotypic slices.** (**A**) Schematic representation of the NVU. Brain vessels are composed of endothelial cells joined together by TJ proteins namely ZO-1, claudin-5 and occludin but also by adherens junctions (AJs) with cadherin/catenin complex. Occludin and claudin-5 are membrane proteins responsible for junction formation and ionic transport. ZO-1 and catenin bind membrane proteins and actin. ZO-1 is responsible for the presence of TJ proteins at the membrane. Endothelial cells are surrounded by end-feet of astrocytes and by pericytes, both essential for the maintenance and regulation of the NVU. Neurons and microglia are also present in the NVU. Many transporters are present at the BBB, including P-glycoprotein (P-gp) and Glucose transporter-1 (Glut-1). All transporters are involved in nutrient passage and brain detoxification, and play an important role in the pharmaco-resistance. (**B**) Blood vessel (laminin- red) and astrocyte (GFAP- green) staining in control slices, or 24 hours after treatment with bicuculline or kainate. Arrows represent astrocyte end-feet. Scale bar: 50 μm. (**C**) Quantification of western blot for claudin-5, occludin and ZO-1, 24 hours after bicuculline or kainate seizures. Results are expressed in percent of control. ** *p*<0.01 (**D**) Photomicrographs of ZO-1 (green) and laminin (red) staining in control slices and 24 h after bicuculline or kainate *in vitro* seizures. Scale bar: 10 μm.

The NVU is mainly composed of endothelial cells characterized by limited transport due to the presence of transporters and TJs. In the brain microvasculature, cells surrounding the capillaries include astrocytes and pericytes. These cells play strategic roles in both formation and maintenance of the NVU, and also in neurovascular coupling [1,9,28-30] (Figure 2A). The presence of astrocyte end-feet along and surrounding blood vessels was demonstrated by GFAP staining in control organotypic cultures (Figure 2B, arrows). The presence of these cells around blood vessels is modified in pathological conditions. Indeed, we observed a reduction of astrocyte end-feet 24 h after kainate treatment. However, after seizures without lesions, end-feet appeared intact (Figure 2B, arrows).

The main characteristic of the NVU is the presence of TJ proteins which scaffold the junctions between endothelial cells. These proteins are essential for a high transendothelial electrical resistance (TEER) (Figure 2A) [29,31]. The three important TJ proteins, ZO-1, claudin-5 and occludin, are preserved in culture for several weeks [21,32].

We studied the regulation of ZO-1, claudin-5 and occludin 24 h post-seizure induced by either kainate or bicuculline. In the kainate model, Western blot analysis revealed a significant down-regulation only for ZO-1, *p*<0.01. In the bicuculline model the expression of the three main TJ proteins was not affected (Figure 2C). The staining of ZO-1 and laminin revealed a regular staining of the TJ proteins along blood vessels in control slices. Similar staining was observed 24 h post-bicuculline seizures. However, at 24 h post-kainate seizures, ZO-1 staining was absent along blood vessels (Figure 2D). This experiment on OHCs showed that NVU remodeling is dependent on severity of neuronal damage induced by epileptiform stimuli.

### OHCs are accessible for molecule screening in the culture medium

**Figure 3:**
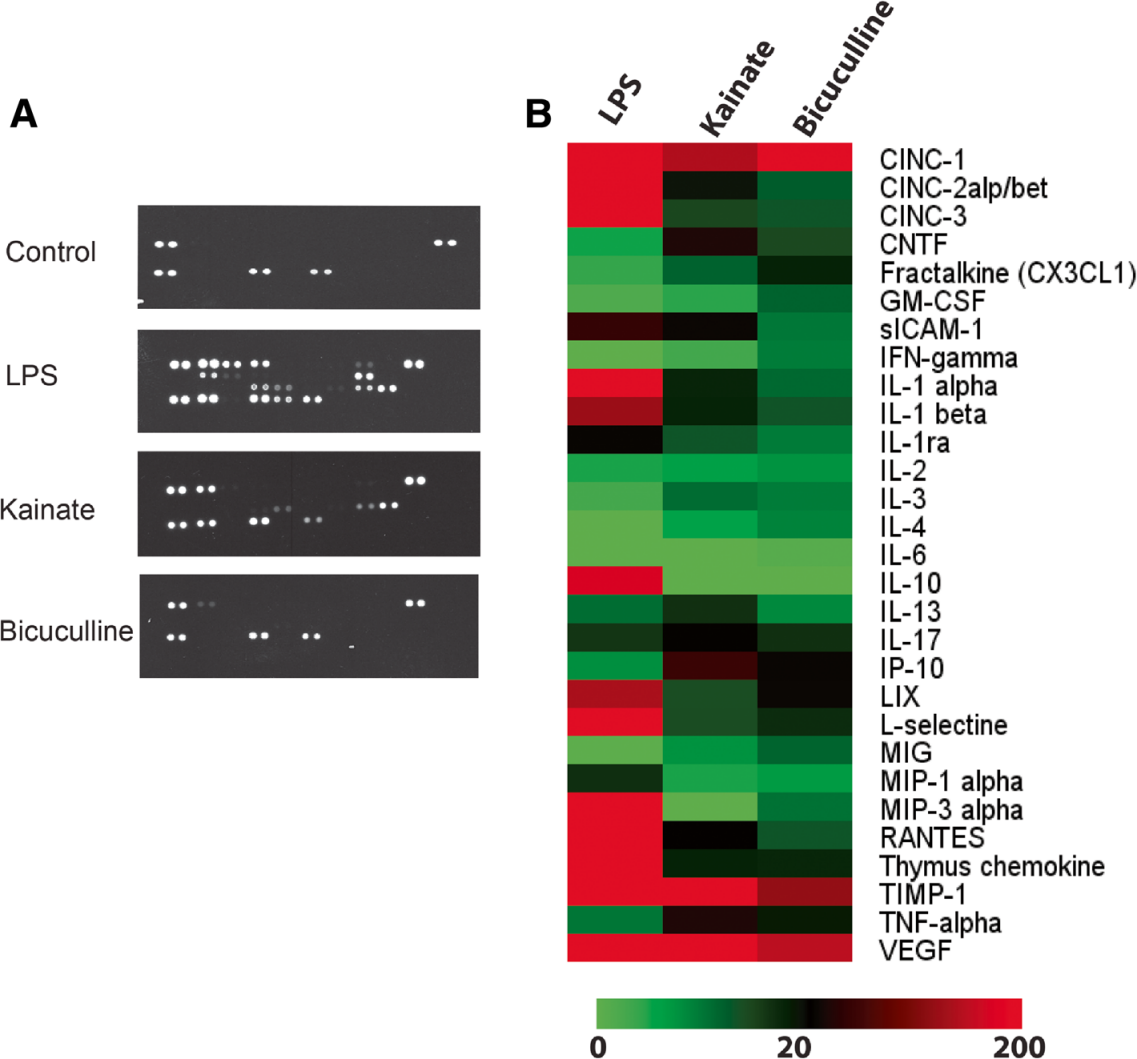
**Organotypic slices are accessible for molecular screening in culture medium.** (**A**) Membranes representing the cytokine micro-array performed on culture medium 24 h after LPS, bicuculline or kainate treatment. Control corresponds to the culture medium of organotypic slices without treatment. (**B**) Heatmaps representing the optical density for each cytokine. Green represents low expression whereas red corresponds to high expression.

The tissue or culture medium from OHCs can be analysed by molecular screening techniques. Due to differences in branching and the regulation of NVU between two seizure models, we can expect differences in the secretion and release of angiogenic factors or cytokines. With a protein array, we evaluated the levels of secreted cytokines 24 h after seizures induced by kainate or bicuculline. As negative and positive controls, we used the medium of non-treated slices and the medium of OHCs treated with LPS. In the medium from non-treated slices, we found only 2 cytokines: VEGF and metallopeptidase inhibitor-1 (TIMP)-1 while 24 h after LPS treatment, many additional cytokines were secreted into the culture medium. After seizure induction, we observed different patterns between kainate and bicuculline models. In kainate model, cytokines present in the culture medium indicated an inflammatory process, confirming previous results [27,33]. In contrast, 24 h after bicuculline seizures, cytokine patterns were identical to those in control slices (Figure 3A). These differences in cytokine profiles were confirmed by heatmap analysis. We focused in more detail on cytokines known to be involved in vascular remodeling by inducing angiogenesis or increased BBB permeability. VEGF, IL-1β, IL-1α, IL-6, IL-13 and also TNF-α are pro-angiogenic, while IFN-γ, IL-1β and VEGF can also participate in BBB permeability (Table 1). Most of these cytokines, including IL-1α, IL1-β but also VEGF, are present at higher levels in the kainate compared to bicuculline model (Figure 3B).

**Table 1:** Role of cytokines in inflammation, angiogenesis and BBB permeability

**Cytokines**	**Others name**	**Inflammation**	**Angiogenesis**	**BBB disruption**	**References**
**CINC-1**	CXCL-1 or Gro-a	**Pro**	**Pro**	**/**	[34]
					[35]
**CINC-2**	CXCL-3 or Gro-g	**Pro**	**Pro**	**/**	[35]
					[36]
**CINC-3**	CXCL-2 or Gro-B	**Pro**	**Pro**	**/**	[35]
					[37]
**CNTF**		**Pro**	**/**	**/**	[38]
**Fractaline**	CX3CL-1	**Pro**	**Pro**	**/**	[39]
					[40]
**GM-CSF**		**Pro**	**Pro**	**Pro**	[40]
					[41]
					[42]
**S-ICAM-1**	CD54	**/**	**/**	**/**	
**IFN-g**		**Pro**	**Anti**	**Pro**	[43]
					[41]
					[44]
**IL1-a**		**Pro**	**Pro**	**Pro**	[43]
					[45]
					[46]
**IL1-b**		**Pro**	**Pro**	**Pro**	[45]
					[47]
					[48]
**IL1-ra**		**Anti**	**Anti**	**/**	[49]
**IL-2**		**Pro**	**Pro**	**Pro**	[50]
					[51]
**IL3**			**Pro**	**/**	[52]
**IL4**		**Pro**	**Anti**	**Pro**	[50]
					[51]
					[35]
**IL6**		**Pro and anti**	**Pro**	**Pro**	[35]
					[48]
					[53]
**IL10**		**Anti**	**Anti**	**/**	[54]
**IP-10**	CXCL10	**Pro**	**Anti**	**/**	[35]
**LIX**	CXCL5	**Pro**	**Pro**	**/**	[35]
**L-selectine**	CD62L	**/**	**/**	**/**	
**MIG**	CXCL9	**Pro**	**Anti**	**/**	[35]
**MIP-1a**	CCL3	**Pro**	**/**	**/**	[35]
**MIP-3a**	CCL20	**Pro**	**/**	**/**	[55]
**RANTES**	CCL5	**Pro**	**Anti**	**Pro**	[56]
**TIMP-1**		**/**	**Anti**	**/**	[57]
**TNF-a**		**Pro**	**Pro**	**Pro**	[48]
					[35]
**VEGF**		**/**	**Pro**	**Pro**	[58]

### Protein analysis in tissue

Angiogenesis and BBB permeability are hallmarks of VEGF/VEGFR-2 activation [59]. Their overexpression in the epileptic focus after experimental seizures suggests that the VEGF/VEGFR-2 system is a logical new target for refractory epilepsies [21,22,60,61]. In the kainate model, we demonstrated an upregulation and activation of VEGF/VEGFR-2 signaling, leading to the downregulation of ZO-1 [21]. The lower level of VEGF in the culture medium of OHCs after bicuculline treatment suggested a different regulation of angiogenic factors that does not trigger a loss of TJ proteins.

**Figure 4:**
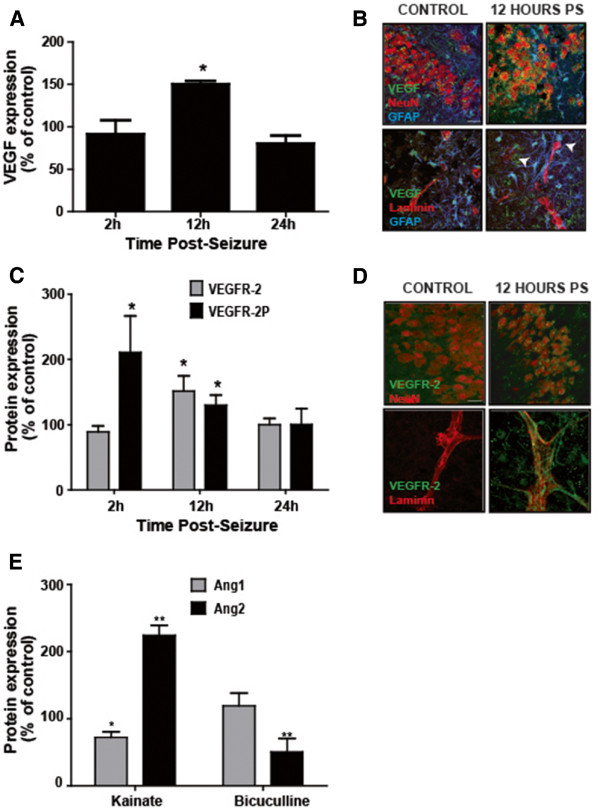
**Organotypic slices are accessible for analysis of proteins in tissue.** (**A**) Analysis of VEGF expression by Western blot 2, 12 and 24 h post-bicuculline treatment. Results are expressed in percent of control, * *p*<0.05. (**B**) Immunostaining of VEGF (green), NeuN or laminin (red) and GFAP (blue) in control slices or 12 h post-bicuculline seizures (PS). VEGF is expressed in neurons and in astrocytes around blood vessels (arrows), Scale bar 10 μm. (**C**) Analysis of VEGFR-2 expression and activation by Western blot 2, 12 and 24 h post-bicuculline treatment. Results are expressed in percent of control, * *p*<0.05. (**D**) Immunostaining of VEGFR-2 (green), NeuN or laminin (red) in control slices or 12 h post-bicuculline seizures. VEGFR-2 is expressed in neurons and along blood vessels 12 h after bicuculline treatment. Scale bar 10 μm. (**E**) Analysis of Ang1 and Ang2 expression by Western blot 24 h post-bicuculline or kainate treatment. Results are expressed in percent of control, * *p*<0.05, ** *p*<0.01.

Western blotting and immunostaining demonstrated an overexpression of VEGF only 12 h after bicuculline application, *p*<0.05 (Figure 4A). Increased VEGF was observed in astrocytic end-feet and surrounding blood vessels (arrows, Figure 4B). We next studied the expression and activation of VEGFR-2. An increase in VEGFR-2 expression was also detected 12 h post-seizure, *p*<0.05. However, the activation of VEGFR-2, measured by the phosphorylation of the receptor, was visible at 2 and 12 h post**-**seizure, *p*<0.05 for both (Figure 4C). The immunostaining revealed an overexpression of the receptor in neurons but more particularly in blood vessels (Figure 4D). After bicuculline-induced seizures, we observed a similar upregulation of the VEGF/VEGFR-2 system.

To understand differences in the regulation of tight junctions between the two seizure models, we focused on the angiopoietin system, composed of angiopoietin-1 and 2 (Ang1 and Ang2). These two proteins have opposite effects on the BBB integrity; Ang1 is involved in the maturation of blood vessels and participates in the BBB integrity, whereas Ang2 appears in early stages of the angiogenesis and disrupts the BBB [62-65]. We decided to study the regulation of these two proteins 24 h after either kainate or bicuculline seizures, a time-point where ZO-1 is downregulated in the kainate model only. By Western blotting, we observed the same level of Ang1 as in control slices but a significant downregulation of Ang2 following bicuculline seizures, *p*<0.01. However, after kainate seizures, Ang1 expression was significantly lower than in control slices, *p*<0.05, whereas Ang2 was significantly upregulated, *p*<0.01 (Figure 4E). These results suggest that angiopoietin system could play an important role in the regulation of TJ proteins after epileptic seizures.

### Drug testing and molecular screening

**Figure 5:**
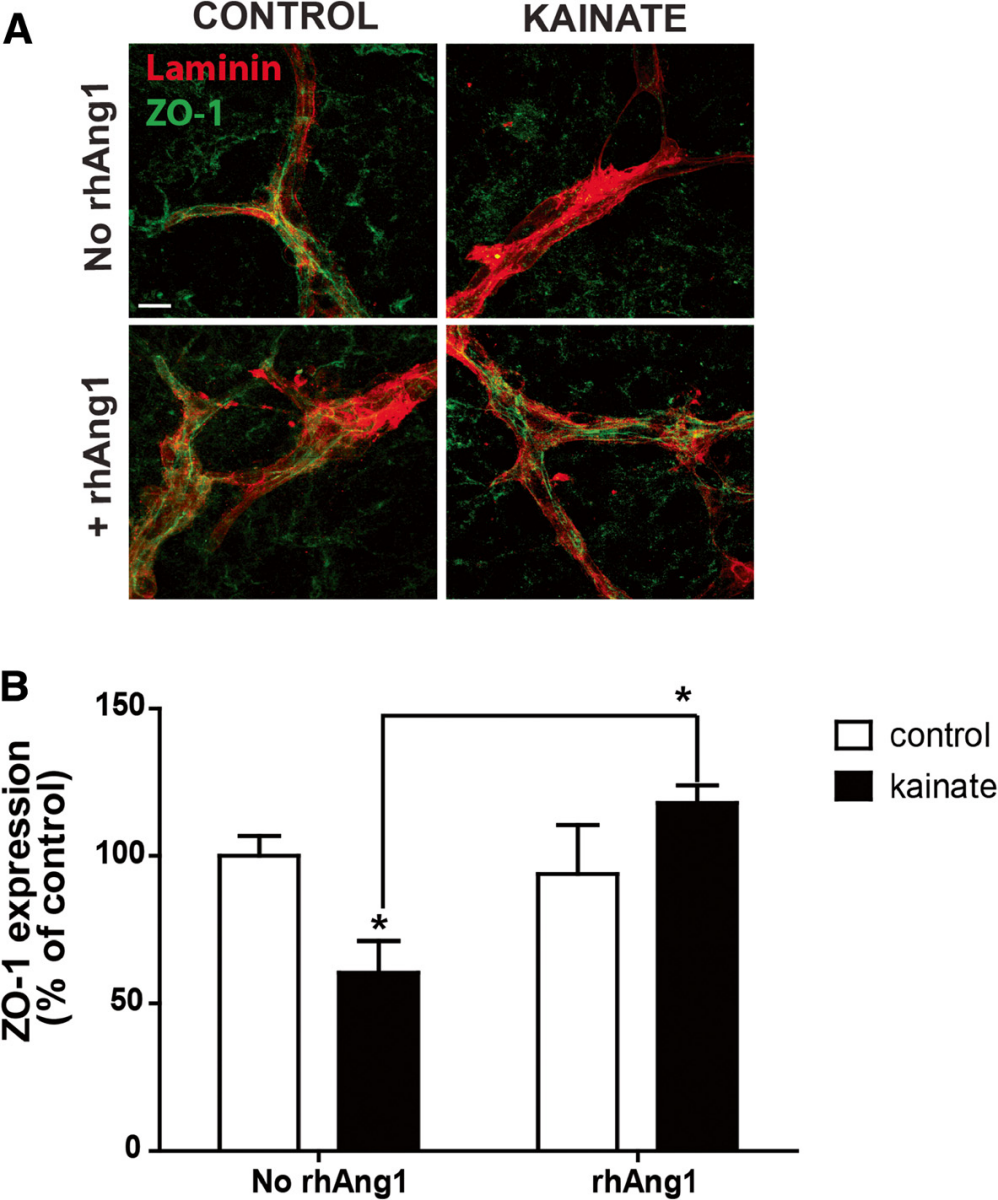
**Organotypic slices are accessible for drugs testing.** (**A**) Immunostaining of ZO-1 (green) and laminin (red) in control slices and 24 h post-kainate seizures with or without rh-Ang1 application 4 h post-seizures. Rh-Ang-1 repaired the loss of ZO-1 (green) in blood vessels (laminin, red). Scale bar: 50 μm (**B**) Analysis of Zonula occludens-1 expression by Western blot 24 h post-kainate treatment with or without rhAng1 application. Results are expressed in percent of control, * p<0.05.

Due to their easy accessibility, OHCs are excellent tools for pharmacological and biochemical assays, including drug screening for BBB protective compounds that could improve the treatments for ischemic or traumatic injuries [66,67]. We have already demonstrated the use of neutralizing antibodies in OHCs. We neutralized VEGF with an anti-VEGF antibody (bevacizumab) that prevents VEGF binding to its receptor. Despite the thickness of the culture plus insert at around 150 μm, the addition of this neutralizing antibody abolished both the down-regulation of ZO-1 protein and increased vascularisation induced by *in vitro* seizures [21]. Since in this study a deregulation of the Ang proteins was observed only in the model where ZO-1 was also downregulated, we tested the effect of recombinant Angiopoietin-1 (rhAng-1) applied to the culture medium. To determine if we can restore ZO-1 expression after kainate seizures, rhAng1 was added to the culture medium 4 h post-seizures. In control slices treated with rhAng1, the staining pattern of ZO-1 along blood vessels did not change compared to control slices. Treatment with rhAng1 after kainate seizures restored the presence of ZO-1 protein (Figure 5A). Western blot analysis confirmed the above results, showing a significant increase of ZO-1 expression after rhAng1 addition, *p*<0.05, and confirming that Ang1 plays an important role in the restoration of BBB integrity (Figure 5B).

## Discussion

Due to the presence of all cell types and their interactions, preservation of TJs between endothelial cells, as well as BBB carriers and transporters, brain slices provide a complete *ex vivo* model of the NVU, although in the absence of blood flow. For example, we have observed that NVU alterations in OHSc, including increased vascularisation and TJ disassembly, were similar to those reported in human *in vivo* focal epilepsy. Other cell types present at the BBB can be studied in OHCs. Indeed, a recent paper focused on the intact functions of NVU in OHCs, showing that calcium signaling can be investigated in astrocyte end-feet and that the contractile properties of pericytes, necessary for vasomodulation, are conserved for weeks in culture [11,68]. Moreover, microglia and neurons are also present in the NVU, but the role of microglia in NVU regulation is still not clear and poorly studied in organotypic cultures. The only link between microglia and blood vessels was the presence of active microglia surrounding blood vessels in retinal organotypic cultures [69]. Finally, the presence of transporters such as glucose transporter−1 and P-glycoprotein on brain endothelial cells in organotypic slices has been previously documented. Moreover, it has been shown that the transport function of P-gp, involved in the pharmacoresistance of several neurological diseases, is still preserved in organotypic slices [30].

Organotypic slices are also useful for testing the ability of different drugs to affect/protect the NVU, such as inhibitors of signaling pathways and neutralizing antibodies [21]. In this study we showed that NVU integrity was restored by the application of recombinant Ang1. BBB dysfunction in diverse CNS disorders, including epilepsy, Alzheimer’s disease and ischemia, is in part due to the loss of AJ or TJ proteins along microvessels [21,22,70,71].

However, a limitation of organotypic cultures is the lack of tools to estimate BBB permeability, which is altered in several brain pathologies [22,72-74]. Staining for serum protein leakage or the measurement of TEER *in vitro*[75] is not possible in this model. To counteract this issue, a co-culture model of endothelial cells and brain slices has been developed in which Duport and colleagues showed that the BBB permeability can be evaluated by microdialysis [76].

In the past decade, alterations in the neuro-vasculature have been shown to be important in many CNS diseases, including glioma, stroke, Alzheimer’s disease and epilepsy [2-5,77]. However the mechanisms of NVU dysregulation are still unknown in several pathologies. Using organotypic slice cultures to study NVU embedded in the microenvironment of anatomically organized parenchymal cells and maintaining many important physiological functions, will undoubtedly facilitate future studies on mechanisms and impact of pathological conditions on NVU remodeling as well as its role in disease processes.

## Abbreviations

AJs: Adherent junctions; Ang1: Angiopoeitin-1; Ang2: Angiopoeitin-2; BBB: Blood–brain Barrier; NVU: Neuro-vascular Unit; OHCs: Organotypic hippocampal cultures; rhAng1: Recombinant humain Angiopoeitin-1; rrVEGF: Recombinant rat VEGF; TEER: Transendothelial electric resistance; TJs: Tight junction proteins; TLE: Temporal lobe epilepsy; VEGF: Vascular endothelial growth factor; VEGFR-2: Vascular endothelial growth factor receptor-2; ZO-1: Zonula occludens-1.

## Competing interests

The authors declare that they have no competing interests.

## Authors’ contributions

MMB, FdB, MLN designed research. FdB performed organotypic hippocampal slices preparation. MMB performed immunostaining and quantified vascularization. MMB and MLN wrote the paper. All authors read and approved the final manuscript.
